# Ring finger protein 125, as a potential highly aggressive and unfavorable prognostic biomarker, promotes the invasion and metastasis of human gallbladder cancers *via* activating the TGF-β1-SMAD3-ID1 signaling pathway

**DOI:** 10.18632/oncotarget.18180

**Published:** 2017-05-25

**Authors:** Zhong-Yan Liu, Jin Cao, Jing-Tao Zhang, Guo-Li Xu, Xin-Ping Li, Fang-Tao Wang, Kamar Hasan Ansari, Hassan Mohamed, Yue-Zu Fan

**Affiliations:** ^1^ Department of Surgery, Tongji Hospital, Tongji University School of Medicine, Tongji University, Shanghai 200065, P.R. China

**Keywords:** gallbladder neoplasm, ring finger protein 125, metastasis, prognosis, signaling pathway

## Abstract

Human gallbladder cancer (GBC) is a lethal aggressive malignant neoplasm. Identification of potential molecular biomarkers and development of targeted therapeutics for GBC patients is very necessary. In this study, we firstly investigated the correlation between ring finger protein 125 (RNF125) expression and the metastasis and prognosis of GBC, and the underlying molecular mechanism. RNF125 expression in a cohort of GBC tissues was examined; its correlation with clinicopathological and prognostic factors of GBC patients was analyzed. Moreover, the metastasis-related difference expressed genes in highly and lowly aggressive GBC cell lines were identified; and the influence of RNF125 knockdown on the metastatic phenotypes and characteristic EMT markers in highly aggressive GBC NOZ cells was detected. Furthermore, the underlying molecular mechanism of RNF125 effect was explored. The results showed that RNF125 was highly expressed in GBC tissues and related with aggressive characteristics such as Nevin stage (*P* = 0.041) etc. and unfavorable prognosis of GBC patients (*P* = 0.023, log-rank test). And, RNF125 was proved to a positive metastasis-related gene *in vitro*. RNF125 knockdown inhibited the invasion and migration, enhanced the adhesion, upregulated E-cadherin and β-catenin expression, and downregulated vimentin and N-cadherin expression (all *P* < 0.001) of NOZ cells *in vitro*. RNF125 promoting effect on GBC tumor progression was identified to relate with the activation of TGF-β1-SMAD3-ID1 signaling pathway. These findings firstly confirm that high RNF125 expression is related with aggressive characteristics and unfavorable prognosis of GBC patients; RNF125 promotes the invasion and metastasis of human GBCs *via* activating the TGF-β1-SMAD3-ID1 signaling pathway.

## INTRODUCTION

Human gallbladder cancer (GBC), a lethal aggressive malignant neoplasm, is the most common malignancy of the biliary tract, the 5th or 6th common malignant neoplasm of the digestive tract and the leading cause of cancer-related deaths in West countries and China [1–4]. Despite significant breakthroughs in improving early diagnosis of the disease, prognosis of the patients is still very poor. Especially, highly aggressive GBC is a considerable clinical problem not only due to diagnostic delay, dismal results of surgical resection and chemo-radiotherapy for the disease, but also due to the complexity of targeting the elusive metastatic phenotypes [4, 5]. Therefore, identification of special biological behaviors, further understanding the mechanism of metastasis and development of potential targeted interventions for GBCs is of very significant, and remain challenging [4–7]. Recent developments in molecular biomarkers, targeted therapeutics, directed against key signal pathways in GBCs appear promising [4, 5, 8–12]. However, specific molecular biomarkers for invasion and metastasis of GBC, which can predict the patient prognosis and the response of targeted therapeutic, are very few and not thoroughly elucidated.

The ubiquitin-proteasome system (UPS) plays important roles in the control of numerous cellular processes, including cell-cycle progression, signal transduction, transcriptional regulation, receptor downregulation, and endocytosis. Ubiquitination is a reversible biochemical process which usually involves three steps and is mediated or participated by ubiquitin activating enzyme (E1), ubiquitin-conjugating enzyme (E2), ubiquitin ligase (E3), and 26s or 30s proteasome that attaches ubiquitin to substrate proteins in order to regulate above multiple cellular functions in UPS [13]. Multiple rounds of ubiquitination result in substrate polyubiquitination that can target proteins for proteasome-dependent destruction. Dysfunction in several ubiquitin-mediated processes has been shown to cause many pathological conditions including malignant transformation [13]; and, growing evidence has demonstrated that ubiquitination is associated with the progression of cancer [14]. There are more than 600 E3 ligases in humans, some of which regulate the expression of tumor-suppressor or tumor- promoting proteins. RNF125 is an E3 ubiquitin-protein ligase [15, 16]. This gene encodes a novel E3 ubiquitin ligase that contains a RING finger domain in the N-terminus and three zinc-bindings and one ubiquitin-interacting motif in the C-terminus; and the encoded protein may function as a positive regulator in the T-cell receptor-signaling pathway [15, 16]. RNF125 promotes p53 degradation; represses p53 functions including p53-dependent transactivation and growth inhibition [17]. The result of a study using orthotropic implantation mouse model and cDNA microarray analysis indicated that the expression level of RNF125 was significantly different between primary tumors in Stage III colorectal cancer patients with lymph node metastasis and Stage II patients without lymph node metastasis, believing that RNF125 could play an important role in lymph node metastasis of colorectal cancer [18]. Recently, Kim H et al reported that downregulation of RNF125 underlay resistance of melanoma cells to BRAF inhibitors *via* JAK1 deregulation, suggesting that combination therapies targeting both JAK1 and EGFR could be effective against BRAFi-resistant tumors with de novo low RNF125 expression [19]. But, there is so far little report about RNF125 involved in GBC, especially GBC invasion and metastasis. In this study, we firstly investigated the correlation between the expression of RNF125 and unfavorable prognosis of GBC patients, identified the existence of RNF125 in highly and lowly aggressive GBC cell lines using human gene expression microarray analysis and the influence of RNF125 knockdown on invasion-metastasis characteristics, epithelial-mesenchymal transition (EMT), downstream genes and signaling pathways in highly aggressive GBC cells. The result firstly demonstrated that RNF125, as a potential highly aggressive and unfavorable prognostic biomarker, promotes the invasion and metastasis of GBCs *via* activating the TGF-β1-SMAD3-ID1 signaling pathways, and is expected to become a potential targeted therapeutic for highly aggressive human GBCs.

## RESULTS

### RNF125 is highly expressed in human GBC tissues and related with aggression and unfavorable prognosis of GBC patients

**Figure 1:**
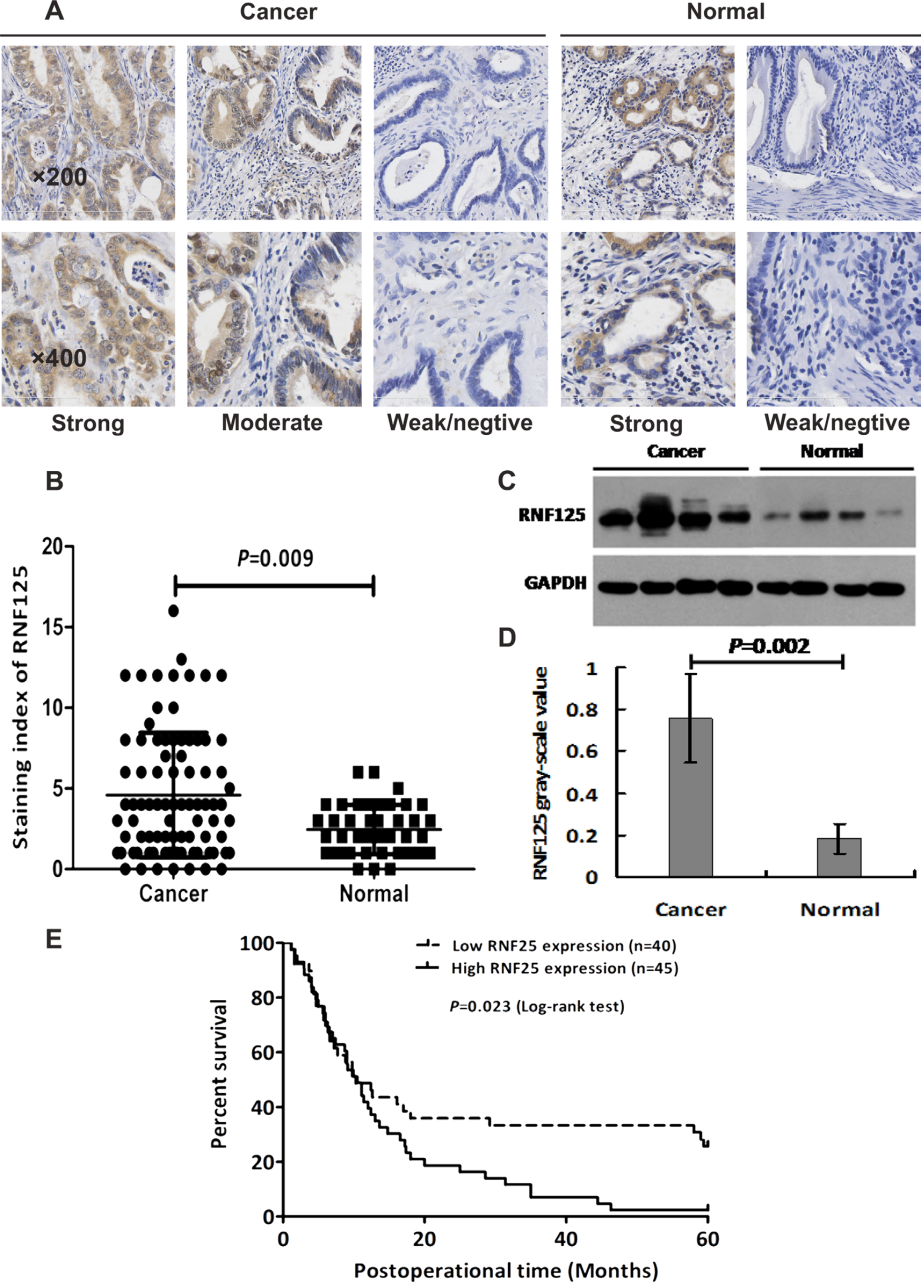
The expression of RNF125 protein in human GBC tissues and Kaplan-Meier survival curves for the GBC patients with high and low RNF125 expression (**A**) The expression of RNF125 protein with yellow or brown staining was predominantly observed in the cytoplasm of cells in GBC and the normal paired tissues. RNF125 protein was highly expressed in GBC tissues *via* immunohistochemistry staining under an inverted light microscope (×200, ×400). (**B**) The staining index (SI) of RNF125 expression in GBC tissues was significantly higher than that of the normal paired samples (*P* = 0.009). (**C**, **D**) The relative grey value of RNF125 protein expression in human GBCs was also significantly higher than that of the normal paired samples by western blotting (*P* = 0.002). (**E**) Kaplan-Meier survival curves for the GBC patients with high and low RNF125 expression. GBC patients with high RNF125 expression had a poorer survival than low RNF125 expression patients (*P* = 0.023, log-rank test).

Immunohistochemistry (IHC) staining and western blotting were used to determine the expression of RNF125 protein in human GBC tissues. As showed in Figure 1, RNF125 expression was predominantly observed in the cytoplasm, partly in the nucleus of the cells (Figure 1A). The staining index (SI) of RNF125 expression in GBC tissues was significantly higher than that of the normal paired samples (*P* = 0.009; Figure 1A and 1B). The expression of RNF125 protein in GBC tissues by western blotting was also significantly higher than that of the normal paired samples (*P* = 0.002; Figure 1C and 1D). And, RNF125 expression was correlated with tumor differentiation (*P* = 0.017), Nevin stage (*P* = 0.042), UICC stage (*P* = 0.021), liver metastasis (*P* = 0.020) and vascular invasion (*P* = 0.016), whereas no correlation was observed with gender, age, tumor location, tumor size, histological type, lymph node metastasis and resection type (all *P* > 0.05; Table 1). Further, the univariate analysis indicated that histological type (*P* = 0.008), tumor differentiation (*P* = 0.000), Nevin stage (*P* = 0.003), UICC stage (*P* = 0.000), liver metastasis (*P* = 0.015), vascular invasion (*P* = 0.000), lymph node metastasis (*P* = 0.000), resection type (*P* = 0.000) and RNF125 (*P* = 0.022) were all prognostic factors for overall survival (OS) in GBC patients; but the multivariate analysis confirmed that only tumor differentiation (*P* = 0.001), Nevin stage (*P* = 0.041), resection type (*P* = 0.020) and RNF125 (*P* = 0.041) were independent prognostic factors for OS in GBC patients (Table 2). Furthermore, GBC patients with high RNF125 expression had a poorer survival than low RNF125 expression patients (*P* = 0.023, log-rank test; Figure 1E). Thus, we believe that RNF125 is highly expressed in human GBC tissues and related with aggression and unfavorable prognosis of GBC patients.

**Table 1:** Correlation between RNF125 and clinicopathological characteristics in GBC patients

Variables	*n*	RNF125 expression		x^2^-value	*P*-value
		Low	High		
Gender					
Male	34	13	21	1.771	0.183
Female	51	27	24		
Age					
≤ 65	41	19	22	0.016	0.898
> 65	44	21	23		
Tumor location					
Bottom	32	19	13	3.125	0.077
Corporis and others	53	21	32		
Tumor size (cm)					
≤ 3.0	44	17	27	2.597	0.107
> 3.0	41	23	18		
Histological type					
Adenocarcinoma	79	39	40	1.261	0.261
Others b	6	1	5		
Tumor differentiation					
High	13	9	4	5.676	0.017 *^a^*
Moderate	36	19	17		
Low	36	12	24		
Nevin staging					
I, II	8	7	1	4.144	0.042 *^a^*
III, IV, V	77	33	44		
UICC staging					
I, II	9	8	1	5.316	0.021 *^a^*
III, IV	76	32	44		
Liver metastasis					
Negative	46	27	19	5.449	0.020 *^a^*
Positive	39	13	26		
Vascular invasion					
Negative	50	29	21	5.835	0.016 *^a^*
Positive	35	11	24		
Lymph node metastasis					
Negative	26	14	12	0.693	0.405
Positive	59	26	33		
Resection type					
R0	37	21	16	2.473	0.116
R1, R2	48	19	29		

^a^ *P* < 0.05, statistically significant;

*^b^* others: mucinous adenocarcinoma, squamous and adenosquamous carcinoma, etc.

**Table 2:** Univariate and multivariate analyses of overall survival rate of GBC patients with Cox proportional hazards model

Variables	Univariate analysis			Multivariate analysis		
	HR	95% CI	*P*-value	HR	95% CI	*P*-value
Gender						
Male vs. female	0.718	0.452–1.140	0.160			
Age						
≤ 65 vs. > 65	1.037	0.656–1.638	0.876			
Tumor location						
Bottom vs. corporis and others	1.554	0.957–2.533	0.075			
Tumor size (cm)						
≤ 3.0 vs. > 3.0	1.444	0.911–2.291	0.118			
Histological type						
Adenocarcinoma vs. others *^b^*	3.303	1.374–7.940	0.008 ^a^			
Tumor differentiation						
High vs. Moderate vs. low	2.980	2.018–4.400	0.000^a^	2.030	1.342–3.066	0.001*^a^*
Nevin staging						
I, II vs. III, IV, V	21.104	2.906–53.243	0.003^a^	8.265	1.091–62.598	0.041*^a^*
UICC staging						
I, II vs. III, IV	8.207	2.556–26.353	0.000^a^			
Liver metastasis						
(−) vs. (+)	1.763	1.115–2.788	0.015^a^			
Vascular invasion						
(−) vs. (+)	2.568	1.614–4.088	0.000^a^			
Lymph node metastasis						
(−) vs. (+)	2.948	1.698–5.119	0.000^a^			
Resection type						
R0 vs. R1, R2	3.025	1.851–4.944	0.000^a^	1.842	1.101–3.083	0.020 *^a^*
RNF125 expression						
Low vs. high	1.755	1.084–2.840	0.022^a^	1.786	1.001–2.554	0.041*^a^*

*^a^ P* < 0.05, statistically significant;

*^b^* others, mucinous adenocarcinoma, squamous and adenosquamous carcinoma;

GBC, gallbladder carcinoma; HR, hazard ratio; CI, confidence interval.

### RNF125 is a potential positive metastasis-related gene *in vitro*

**Figure 2:**
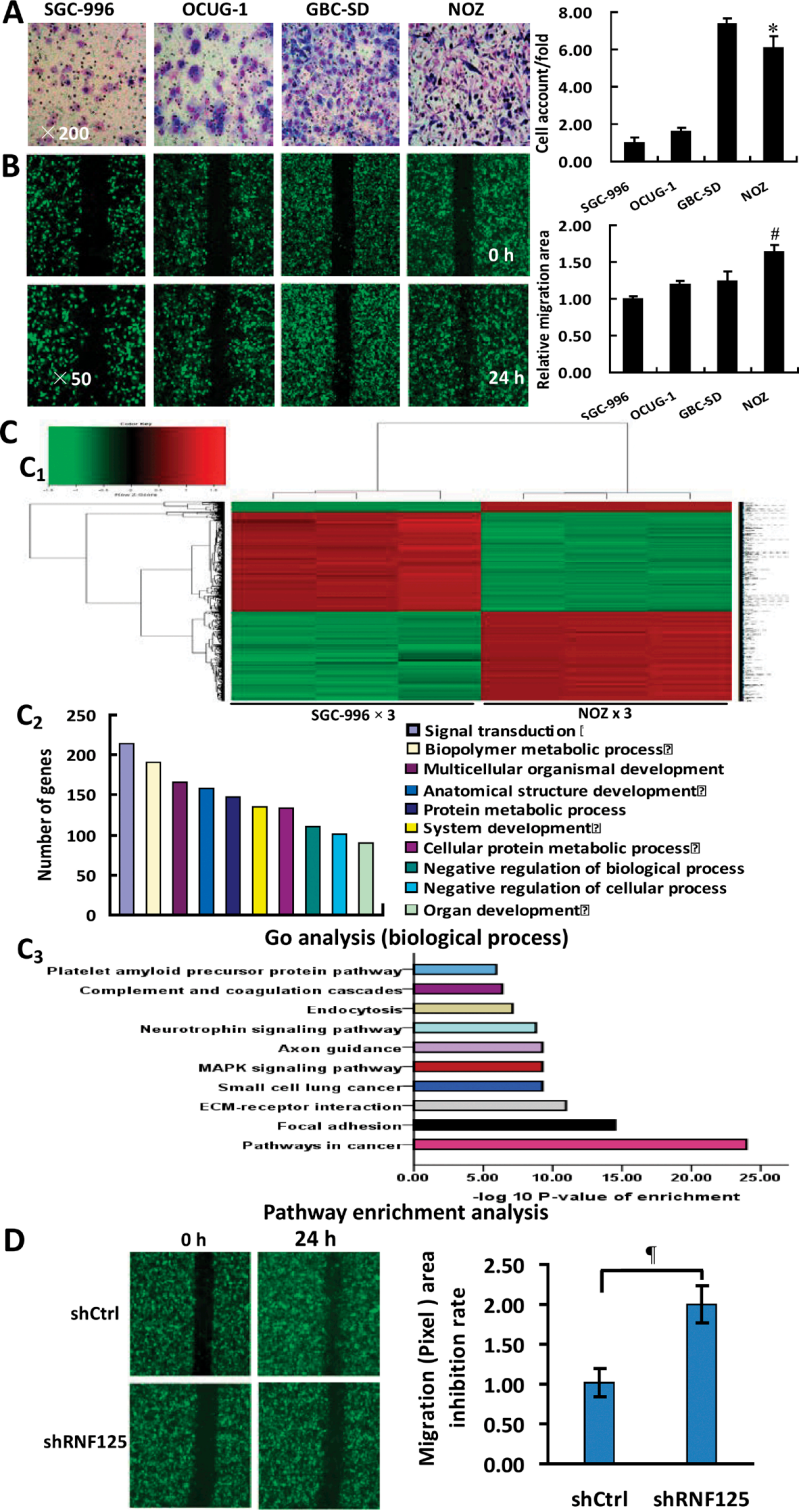
Identification of RNF125 as a positive metastasis-related gene *in vitro* (**A**) Invasive assay: a higher invasive capability (related invasive ability = cell account/found, ×200) was observed in NOZ cells when compared to GBC-SD, OCUG-1 or SGC-996 (*P = 0.017, = 0.000 or = 0.000) cells, with the lowest invasive capability in SGC-996 cells and the highest invasive capability in GBC-SD cells. (**B**) Migration assay: the migration capability (related migration area, ×50) of NOZ cells was significantly higher than that of GBC-SD, OCUG-1 or SGC-996 cells (^#^*P* = 0.006, = 0.002 or = 0.000), with the lowest migration capability in SGC-996 cells. (**C**) Microarray analysis for highly aggressive NOZ and lowly aggressive SGC-996 GBC cells. Heat-map depicting significantly affected genes (red color denotes upregulation and green denotes downregulation) in NOZ cells and SGC-996 cells (C1); gene ontology (GO) analysis based on classification of gene numbers such as biological processes (C2); pathway enrichment analysis for related genes based on the significance probability (C3). (**D**) RNF125 identification *via* knockdown and migration assay with HCS. In 20 metastasis-related, knockdown candidate genes, the migration (Pixel) area in knockdown RNF125 was significantly decreased in NOZ cells when compared to NC (^¶^*P* = 0.025), suggesting RNF125 as a potential positive metastasis-related gene.

To testify whether RNF125 is a highly aggressive GBC gene, we firstly performed the invasion and migration assays for 4 human GBC cell lines (GBC-SD, NOZ, OCUG-1 and SGC-996). As showed in Figure 2, a higher invasive capability and the highest migration capability were observed in NOZ cells when compared to GBC-SD, OCUG-1 and SGC-996 cells (*P = 0.017 and *^#^P* = 0.006, *P = 0.000 and ^#^*P* = 0.002, *P = 0.000 and ^#^*P* = 0.000), with the lowest invasive or migration capability in SGC-996 cells (Figure 2A and 2B). NOZ cell line was thus considered as highly aggressive GBC cell line while SGC-996 cell line as lowly aggressive GBC cell line. Secondly, we performed microarray analysis for highly aggressive NOZ cells and lowly aggressive SGC-996 cells using the GeneChip® PrimeView™ Human Gene Expression Microarray. According to the inclusion criteria, a total of 1048 upregulated genes and 1053 downregulated genes were identified (Figure 2C). The associated heat-map provided an overview of the significantly affected genes (Figure 2C1). Gene ontology (GO) enrichment analysis, categorizing the regulated genes into different sets of biological processes (Figure 2C2), suggested that among the analyzed gene sets, genes related to pathways in cancer, focal adhesion and extracellular matrix (ECM)-receptor interaction pathways were the top three showing the greatest changes in expression, based on significant probability (Figure 2C3). To identify potential aggressive-related gene in NOZ cells, we focused on some genes that might be linked with the metastasis-related genes. Based on this bioinformatics analysis, a total of 39 metastasis-related genes including RNF125 showing 3.0 fold changes of mRNA expression using qRT-PCR analysis in NOZ cells were selected from the microarray data. Lastly, we performed the migration assay with high content screening (HCS) for 20 candidate genes using NOZ cells transfected with shRNA and negative control (NC, shCtrl) to further validate if these genes are metastasis-related genes. The results suggested that among these candidate genes, the metastatic capability of RNF125 showing 1.5 fold changes in migration (Pixel) area was significantly decreased after knockdown in NOZ cells when compared to NC (*P* = 0.025; Figure 2D). Therefore, RNF125 is believed as a potential positive metastasis-related gene.

### Lentivirus-mediated RNF125 knockdown in highly aggressive GBC NOZ cells *in vitro*

**Figure 3:**
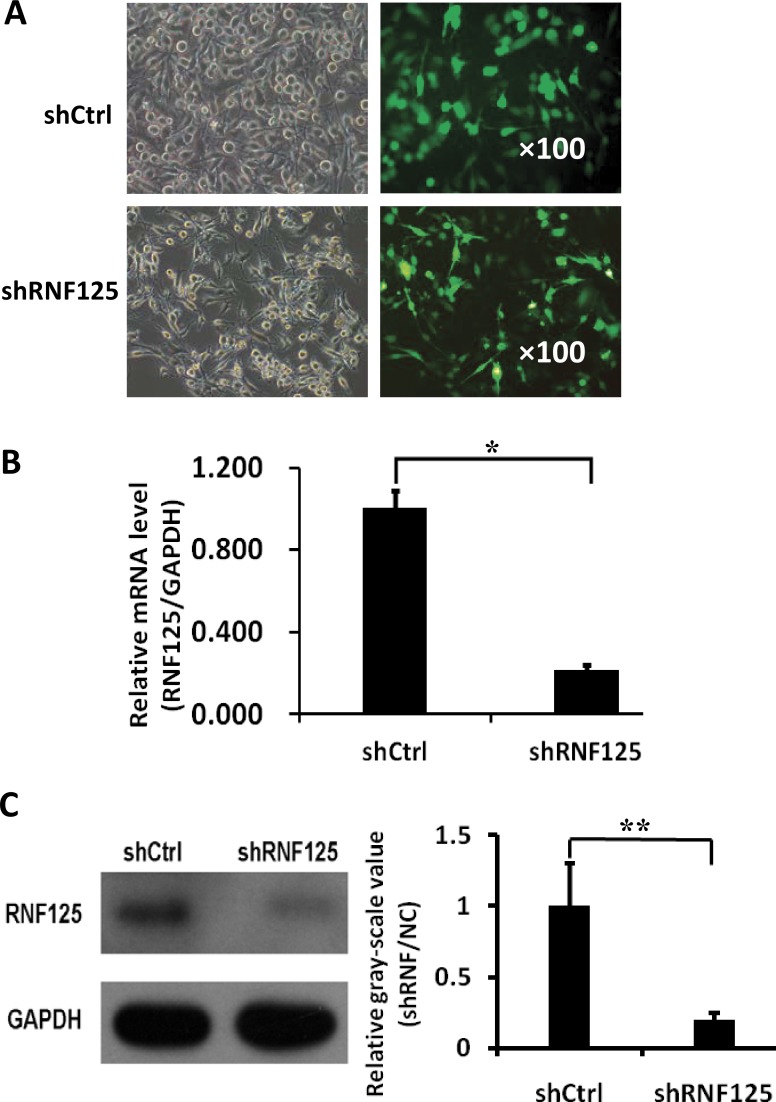
Lentivirus-mediated shRNF125 knockdown in NOZ cells and its qRT-PCR and western blotting confirmation (**A**) The transfection and the greatest knockdown efficiency (80%) of lentivirus shRNA among three shRNAs based on the GFP-expression in NOZ cells as evaluated using a fluorescence microscopy. (**B**) QRT-PCR analysis showed that RNF125 mRNA expression was significantly downregulated by shRNA as compared to shCtrl (*P = 0.000), with 78.3% of knockdown efficiency in NOZ cells. (**C**) Western blotting showed that expression of RNF125 protein was decreased by shRNA when compared to shCtrl (**P = 0.001), thus confirming the knockdown efficiency of shRNF125 in NOZ cells.

In order to analyze the functional roles of RNF125 for GBC cells, three different shRNAs cloned into green fluorescent protein (GFP)-expressing lentiviral vectors were screened in NOZ cells. As showed in Figure 3, the greatest mean knockdown efficiency (80%) shRNA among three shRNAs based on the GFP-expression (Figure 3A) was selected for subsequent experiments. Moreover, qRT-PCR analysis showed that RNF125 mRNA expression was significantly downregulated by shRNA as compared to the negative control shRNA (shRNF125 *vs*. shCtrl, *P = 0.000; Figure 3B), with 78.3% of knockdown efficiency in NOZ cells. Western blotting also confirmed that expression of RNF125 protein was significantly decreased by shRNA when compared to shCtrl (**P = 0.001; Figure 3C) before proceeding with further *in vitro* experiments; thus confirming the knockdown efficiency of shRNF125 in NOZ cells.

### Influence of RNF125 knockdown on invasion, migration, adhesion and EMT of GBC cells *in vitro*

**Figure 4:**
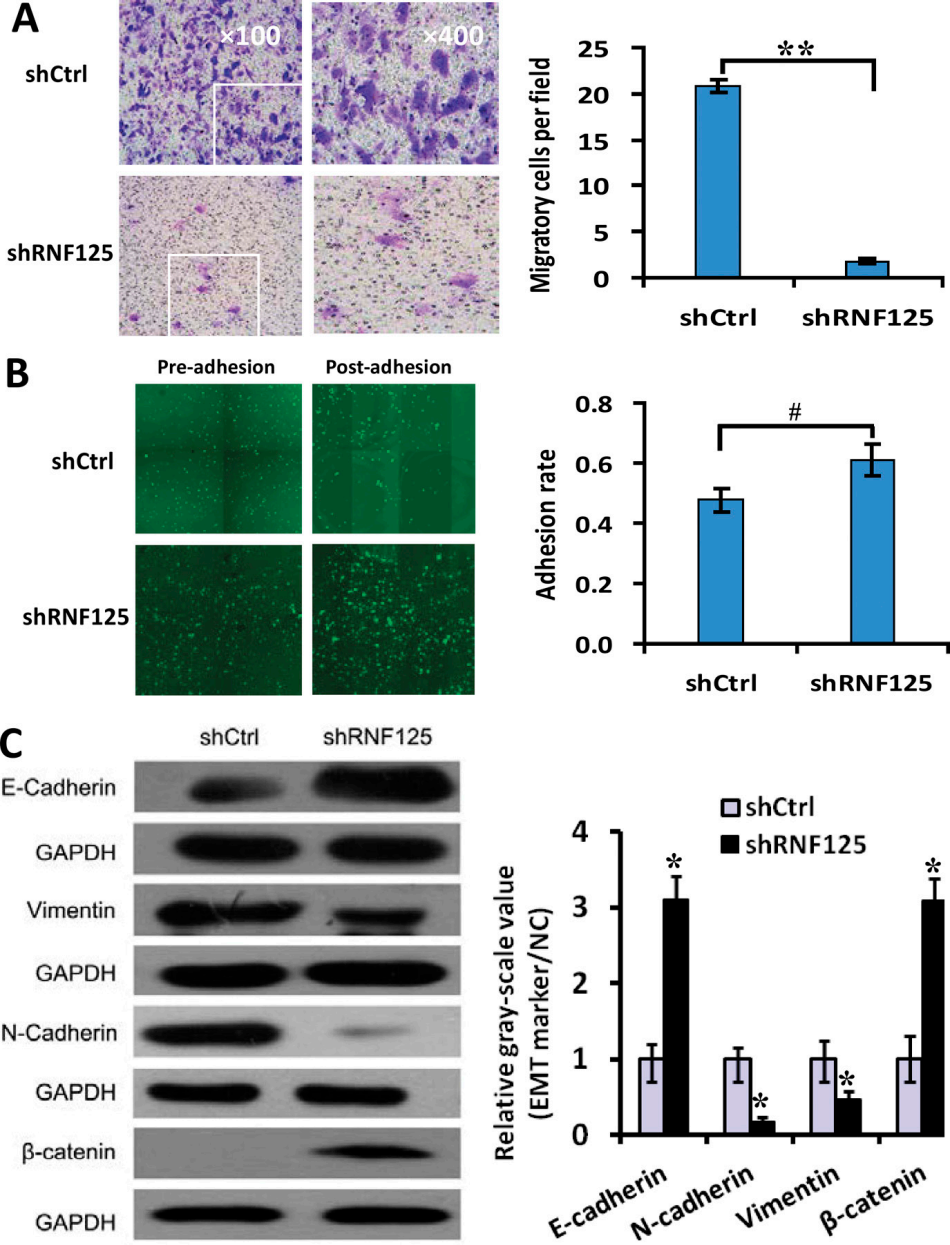
The influence of RNF125 knockdown on the invasion, adhesion and EMT of GBC NOZ cells (**A**) Invasive assay, the invasive cells per field of NOZ cells were dramatically reduced following RNF125 knockdown (shRNF125 *vs*. shCtrl, **P = 0.000). (**B**) Adhesion assay, the adhesion rate of NOZ cells was significantly increased in shRNF125 as compared to shCtrl (^##^*P* = 0.022). (**C**) Western blotting, EMT process was inhibited, i.e., the expression of epithelial marker E-cadherin and β-catenin expression was upregulated, the expression of mesenchymal marker vimentin and N-cadherin was downregulated in shRNF125 as compared to shCtrl (all *P < 0.001).

In order to further testify whether RNF125 is a highly aggressive GBC gene, we assessed the influence of RNF125 knockdown on metastasis-related functions such as invasion and the adhesion of NOZ cells using Transwell invasion and adhesion assays as well as above migration assay with HCS as Figure 2D. After 3 days of lentiviruses infection shRNA, the invasive cells per field of NOZ cells were significantly reduced following RNF125 knockdown (Figure 4A; **P = 0.000); and, the adhesion rate of NOZ cells was significantly increased in shRNF125 as compared to shCtrl (Figure 4B, ^#^*P* = 0.022). These results in combination with the migration assay as Figure 2D suggested that RNF125 knockdown inhibited the invasion and migration functions, advanced the adhesion of NOZ cells. To evaluate if these changes were the result of an EMT, we further detected specific EMT markers using western blotting. RNF125 knockdown for 5 days led to the upregulated expression of the characteristic epithelial marker E-cadherin and β-catenin, and the downregulated expression of the characteristic mesenchymal marker vimentin and N-cadherin (Figure 4C; all *P < 0.001), thus RNF125 knockdown resulted significant inhibition of EMT process. The results indicated that above metastatic phenotype changes associated with shRNA treatment were the results of EMT inhibition. Taken together, RNF125 knockdown inhibited the invasive and migration, and advanced the adhesion of NOZ cells through depressing the EMT process. Therefore, RNF125 is confirmed to be a potential metastasis-related gene.

### RNF125 promotes the invasion and metastasis of human GBCs via activating the TGF-β1-SMAD3-ID1 signaling pathway

**Figure 5:**
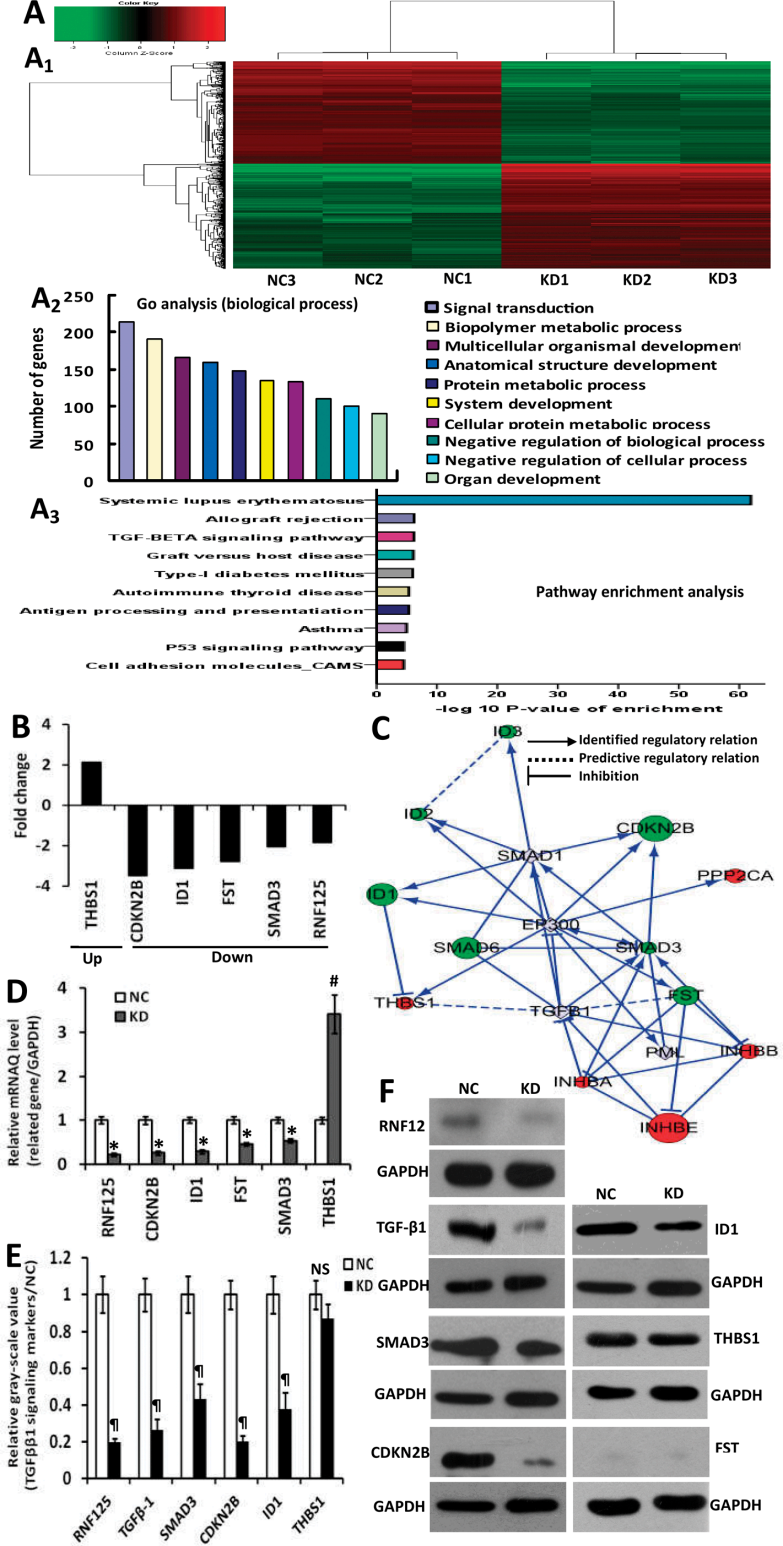
Microarray analysis of NOZ cells transfected with RNF125 or NC, and the construction and confirmation of the TGF-β1-SMAD3-ID1 signaling pathway using the reactome database, qRT-PCR and Western blotting (**A**) Microarray analysis: heat-map depicting significantly affected genes in NOZ cells transfected with RNF125 or NC. A total of 388 upregulated genes and 331 down-regulated genes were identified following RNF125 knockdown (A1); Go analysis (biological process) based on the classification of gene numbers, expression and significance probability (A2) and pathway enrichment analysis for related genes based on significance probability, suggested that among the analyzed gene sets, genes related to TGF-β1 signaling pathway were the top three showing the greatest changes in expression, based on significance probability (A3). (**B**) Fold changes of five putative TGF-β1 pathway genes including SMAD3, CDKN2B, ID1, FST and THBS1 based on the microarray analysis. (**C**) Knowledge-based interactive network for five selected targets in TGF-β1 pathway was constructed using the Reactome database. In gene chip and this network, red denotes upregulated gene, green denotes downregulated gene, while gray denotes system increased related genes. (**D**) The confirmation of the identified target genes using qRT-PCR analysis. The expression of SMAD3, CDKN2B, ID1 and FST mRNAs after RNF125 knockdown in NOZ cells were significantly downregulated (all *P < 0.000), the expression of THBS1 mRNA was significantly upregulated (^#^*P* = 0.011). (**E**, **F**) The confirmation of the identified target proteins using western blotting. The expression of TGF-β1, SMAD3, CDKN2B and ID1 proteins were significantly downregulated in NOZ cells transfected shRNA125 when compared to NC (all ^¶^*P* < 0.05), while no different THBS1 expression and negative FST expression were observed. Thus, TGF-β1, SMAD3, CDKN2B and ID1 have been proved to be the targeted proteins and related molecules of RNF125 downstream TGF-β1 signaling pathway. GAPDH served as a reference control.

**Figure 6:**
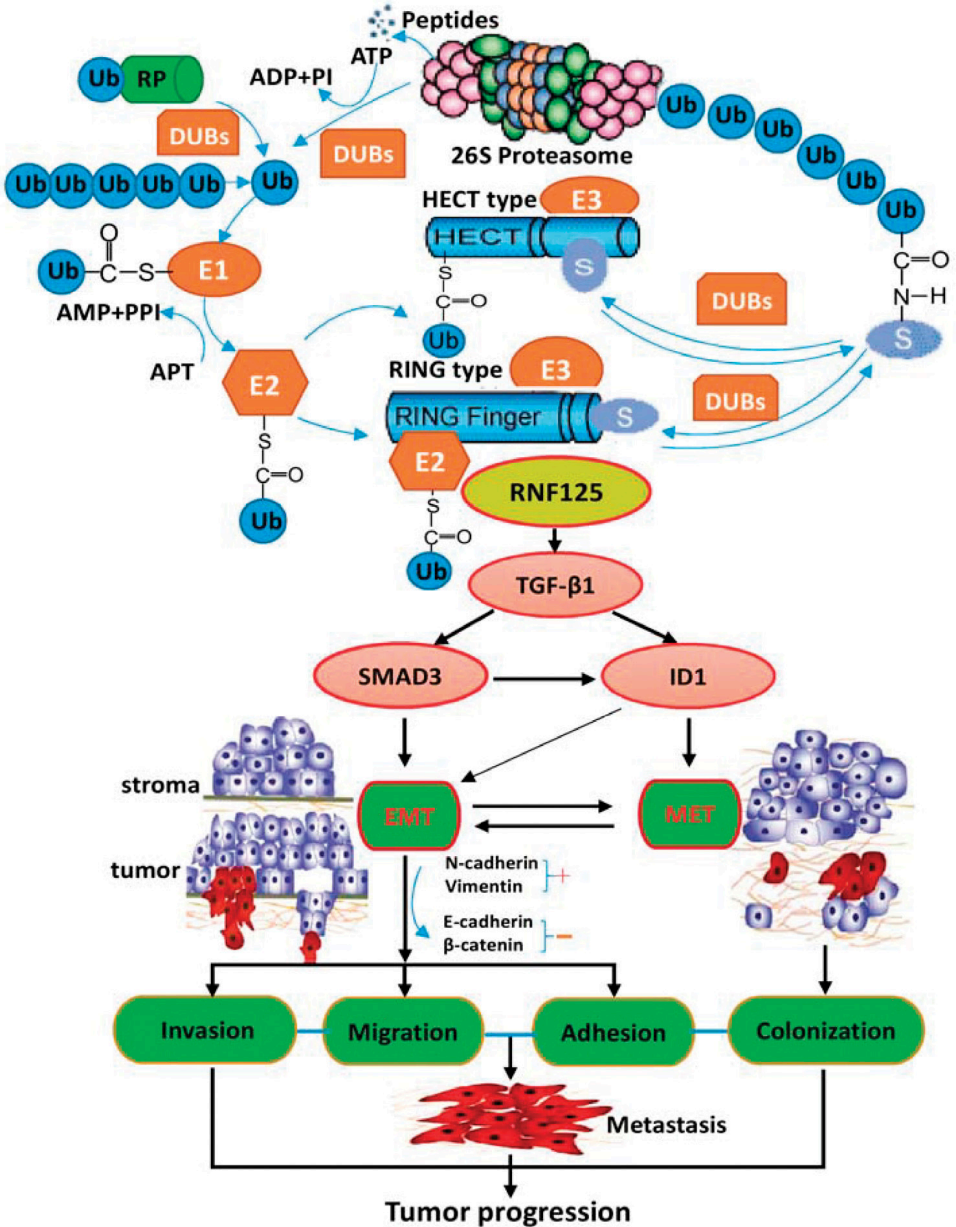
The possible underlying molecular mechanisms of RNF125 effect via activating the TGF-β1-SMAD3-ID1 signaling pathway in cancer progression It appears that high RNF125 expression upregulates TGF-β1, SMAD3 and ID1 expression in RNF125 downstream; on the one hand, upregulated TGF-β1, SMAD3 and ID1 strengthens EMT process by activating TGF-β1-SMAD3-ID1 signaling pathway, accelerating the invasion, migration and adhesion of GBC cells; on the other hand, highly expressed TGF-β1 and ID1 enhances MET process by activating TGF-β1-ID1 signaling pathway, stimulating the colonization of GBC cells; lastly promoting the metastasis and progression of GBC cells. Thus, RNF125 promotes the invasion and metastasis of human GBCs *via* activating the TGF-β1-SMAD3-ID1 signaling pathway.

To explore the potential molecular mechanism of RNF125 effect on the aggression and metastasis of GBC cells, we further performed microarray analysis using NOZ cells transfected with RNF125 and NC (KD *vs*. NC; Figure 5A). Based on the inclusion criteria showing fold changes > 2.0 and *P* value < 0.05, a total of 388 upregulated genes and 331 downregulated genes were identified following RNF125 knockdown. The associated heat-map (Figure 5A1) provided an overview of the significantly affected genes. GO analysis, categorizing the regulated genes into different sets of biological processes, showed that signal transduction was the top one showing the maximum number of genes (Figure 5A2). And, pathway enrichment analysis for related genes suggested that among the analyzed gene sets, twelve genes related to transforming growth factor-β1 (TGF-β1) signaling pathway were the top three showing the greatest changes, based on significance probability of *P*-value 6.54E-07 (Figure 5A3). The TGF-β1 signaling pathway was thus chosen to identify specific potential targets of RNF125. Moreover, based on above significantly affected pathway gene sets and the bioinformatics of microarray analysis, we chosen five genes correlated with TGF-β1 signaling pathway [SMAD family member 3 (SMAD3), cyclin-dependent kinase inhibitor 2B (CDKN2B), inhibitor of differentiation and DNA binding-1 (ID1), thrombospondin1 (THBS1), and follistatin (FST)] showing 2.0 fold changes from the microarray data (Figure 5B). An interaction network was then established using the Reactome database by automatically adding key pathway molecules (Figure 5C). Furthermore, to further validate these five targets as TGF-β1 signaling network markers, we performed qRT-PCR analysis for these related target genes. QRT-PCR analysis showed that the expression of SMAD3, CDKN2B, ID1 and FST mRNAs after RNF125 knockdown in NOZ cells were significantly downregulated (all *P < 0.000), while the expression of THBS1 mRNA were significantly upregulated (^#^*P* = 0.011) (Figure 5D). In view of TGF-β1 as a system increased related gene in gene chip and interaction network, we further performed western blot analysis. Western blotting showed that the expression of SMAD3, CDKN2B and ID1 proteins as well as TGF-β1 protein was significantly downregulated in NOZ cells transfected shRNA125 when compared to NC (all ^¶^*P* < 0.05), whereas no different THBS1 expression and negative FST expression were observed (Figure 5E and 5F). So, TGF-β1, SMAD3, CDKN2B and ID1 have been proved to be the targeted proteins and related molecules of RNF125 downstream TGF-β1 signaling pathway. Considering no correlation between CDKN2B gene and invasion-metastasis feature, took together, these results proposed a putative working network model depicting the tentative mechanism by which RNF125 enhanced GBC invasion, metastasis and progression, lastly leading to a poor prognosis (Figure 6). Thus, we firstly confirm that RNF125 promotes the invasion and metastasis of human GBCs *via* activating the TGF-β1-SMAD3-ID1 signaling pathway.

## DISCUSSION

Human GBC is a highly aggressive malignant tumor with a poor prognosis. Surgical resection, chemotherapy and radiotherapy for the disease are disappointing [1–7]. Clearly, novel adjuvant therapies, potential anticancer agents or molecularly targeted therapeutics in advanced GBC are very necessary *in vivo*. Considering the high aggressive characteristic and confusing developmental mechanisms of human GBCs, many researchers are currently seeking to develop new biomarkers, molecular targets or signaling pathways, such as AEG-1, HIF-1α, SPOCK1, PIK3CA and EGFR molecules, and ErbB, PI3K/AKT, PI3-K/MMPs/Ln-5γ^2^ and EphA2/FAK/Paxillin signaling pathways and their inhibitors [4, 5, 8–12]. In recent years, ubiquitination and UPS composed of E1, E2 and E3 has been paid great attention [13, 14]. Abnormal ubiquitination in several ubiquitin-mediated processes has been demonstrated to cause some pathological conditions including malignant transformation and cancer progression [13, 14]. RNF125, as a little studied E3 ubiquitin-protein ligase, was recently reported to play a promoting role in lymph node metastasis of colorectal cancer [18]; its expression downregulation was correlated with resistance of melanoma cells to BRAF inhibitors [19]. Therefore, the potential effects and possible underlying mechanisms of RNF125 involved in GBC especially GBC invasion and metastasis have aroused our great attention. In this study, we confirmed that high RNF125 expression was correlated with high aggression and unfavorable prognosis of GBC patients; that RNF125 was an invasion-metastasis-related gene in GBC cells *in vitro*; and that RNF125 knockdown inhibited the invasion and migration and enhanced the adhesion of highly aggressive NOZ cells *in vitro*. Thus, we firstly conclude that RNF125 may be a potentially highly aggressive and unfavorable prognostic biomarker in GBCs, and promotes the invasion and metastasis of GBCs.

The EMT is a key process associated with tumor invasion, migration, metastasis and progression [20]. EMT converts polarized epithelial cells to motile mesenchymal cells, operates during embryonic cell layer movements and tumor cell invasiveness; and during EMT, the epithelial markers E-cadherin and cytokeratin are downregulated, while the mesenchymal markers N-cadherin, vimentin and α-smooth muscle actin are upregulated [21]. EMT plays an important role in development and progression of a variety of human cancers [22–25]. In this study, we also detected some specific EMT markers by western blotting to evaluate whether RNF125 affecting metastatic phenotypes of NOZ cells was the result of EMT change. The results indicated that RNF125 knockdown enhanced the expression of E-cadherin and β-catenin, and the adhesion of cells, decreased the expression of vimentin and N-cadherin, inhibited the EMT process, thus leading to above metastatic phenotype changes. It is so believed that RNF125 knockdown inhibits the invasive and migration capabilities and enhances the adhesion capability of NOZ cells through inhibiting the EMT process.

Molecular events displayed by highly aggressive GBCs remain poorly understood. In this study, we further explored the underlying mechanism of RNF125 effect on the aggression and metastasis of GBC cells. The results showed that TGF-β1, SMAD3 and ID1 were significantly downregulated in NOZ cells after RNF125 knockdown, were proved to be the targeted proteins and related molecules of TGF-β1 signaling pathway. TGF-β is a multifunctional growth factor that plays complex roles in the growth, progression and metastatic potential of cancers, is highly expressed in a variety of human cancers and correlated with tumor invasiveness, progression, distant metastasis and poor prognosis [26, 27]. And, TGF-β induces EMT in late-stage cancer, leading to enhance the motility, invasion, migration and metastasis of cancers. Blockade of TGF-β or TGF-β-induced EMT inhibits tumor invasion and metastasis, and is a proven therapeutic approach for metastatic progression in some cancers [28–33]. Thus, TGF-β1 is believed as a key inducer of the EMT promoting tumor metastasis. SMAD3, as a major mediator of TGF-β signaling, activates TGF-β-induced EMT, promotes EMT and tumor invasion [34–36]. The TGF-β/SMAD3 pathway is so crucial in EMT induction due to its multiple downstream effectors, which are capable of repressing E-cadherin and subsequently enabling upregulation of mesenchymal promoters, then promoting the migration and invasion of cancer cells [37–39]. ID1 is also reported to be involved in TGFβ1-induced EMT activation, thus enhancing tumor progression in human cancer cells [40–44]; while inactivation of ID1 by inhibiting ID1 expression *via* berberine has potential therapeutic value against primary and metastatic growth of hepatocellular cancer [45]. It is worth noting that Stankic and colleagues [46] recently identified ID1 as a critical regulator of breast tumor-initiating phenotype and metastatic colonization. During metastatic colonization, ID1 induces the mesenchymal-epithelial transition (MET) in cells that had previously undergone EMT. ID1 is induced by TGF-β specifically in cells that have first undergone EMT, and upregulation of ID1 by TGF-β occurs only in disseminated cancer cells that had initially seeded in a mesenchymal state, suggesting that EMT is a prerequisite for subsequent ID1-induced MET during lung colonization. This complex context-dependent function of ID1 allows breast cancer cells to retain their EMT-cancer stem cell-like mesenchymal phenotype during tumor initiation and metastatic dissemination, and to re-acquire their epithelial character necessary for lung colonization. Clearly, this study strengthens the concept that ID1-mediated phenotypic switching and epithelial-mesenchymal plasticity are crucial at different stages of metastasis [46, 47]. According to these literature reports and our recent study findings, took together, we proposed a putative RNF125 working network model depicting the tentative mechanism by which RNF125 stimulates GBC invasion, metastasis and progression lastly leading to a poor prognosis (Figure 6). It appears that high RNF125 expression upregulates RNF125 downstream gene TGF-β1, SMAD3 and ID1 expression; on the one hand, upregulated TGF-β1, SMAD3 and ID1 strengthens EMT process by activating the TGF-β1-SMAD3-ID1 signaling pathway, accelerating the invasion, migration and adhesion of GBC cells; on the other hand, highly expressed TGF-β1 and ID1 enhances MET process by activating the TGF-β1-ID1 signaling pathway, stimulating the metastatic colonization of GBC cells; lastly promoting the metastasis and progression of GBC cells. Thus, these findings firstly demonstrate that RNF125 promotes the invasion and metastasis of human GBCs *via* activating the TGF-β1-SMAD3-ID1 signaling pathway.

Collectively, the results in present study firstly demonstrate that RNF125 is highly expressed in GBC tissues and correlated with high aggression and unfavorable prognosis of GBC patients; that RNF125, as a potential highly aggressive and unfavorable prognostic biomarker, promotes the invasion and metastasis of human GBCs *via* activating the TGF-β1-SMAD3-ID1 signaling pathway. Obviously, it is necessary to further explore RNF125 effects and underlying mechanisms using animal metastatic xenograft model and targeted signaling pathway inhibitors or potential anticancer agents, so as to provide potential therapeutic targets for human GBCs.

## MATERIALS AND METHODS

### Patients and tissue specimens

This study was conducted in accordance with the ethical standards, the Declaration of Helsinki and the official recommendations of the Chinese Community Guidelines, and was approved by the Ethics Committee and the Institutional Review Board at the Tongji Hospital. Written informed consent was obtained from all patients. A total of 148 gallbladder tissue specimens including 128 paraffin-embedded specimens comprised 85 GBC and 43 adjacent normal paired specimens, and 10 fresh GBC and 10 adjacent normal paired specimens were obtained from patients who underwent operation and were histopathologically confirmed at the Tongji Hospital, Tongji University School of Medicine between August 2006 and September 2011. All patients had not received chemotherapy or radiotherapy before surgery. Curative resection (R0 resection) was defined as no residual tumor status, whereas microscopic (R1 resection) and macroscopic residual tumor (R2 resection) was defined as non-curative resection. To reduce effects directly related to surgery, patients who died within one month after surgical resection were not included. Two independent pathologists blinded to the patients' clinical status verified diagnoses of these GBC samples. According to WHO criteria and the Nevin stage system, detailed clinicopathological and follow-up data were collected from the patient's medical records and completed by a telephone survey, routine visit record and address. Clinical outcome was followed from the date of surgery to the date of death or until the end of September 30, 2011. Cases lost during follow-up were regarded as censored data for the survival analysis. A large cohort of 85 patients from whom GBC (a rare tumor) specimens were obtained had been followed up. The median follow-up period for all patients was 19.7 (range, 1–60) months. The 5-year OS rate was 12.9% (11/85). Demographic and clinicopathological data are summarized in Table 1.

### IHC staining *in vivo*

IHC for paraffin-embedded GBC and the adjacent normal paired specimens were performed as previously described [7]. The sections were incubated in order with primary anti-rabbit RNF125 (1:200; Sigma, USA), secondary anti-rabbit IgG (Maixin, China), Vulcan Fast Red Chromogen Kit 2 (Maixin), and DAB solution according to the manufacturer's instructions. Negative controls were established by replacing the primary antibody with PBS in all samples. Evaluation for RNF125 was scored with IHC using a semi-quantitative system with the staining index (SI). SI was calculated using the following formula: (positive cell percentage score) (staining intensity score). The positive cell percentage was scored from 0 to 4 as follows: 0 (no positive cells), 1 (1%-25%), 2 (26%-50%), 3 (51%-75%) and 4 (76%-100%). The staining intensity was scored from 0 to 3 as follows: 0 (negative), 1 (weak), 2 (moderate), and 3 (strong). 3 of SI score were used to distinguish between low (IS ≤ 3) and high (IS > 3) protein expression. In order to score the stains, five random fields of each section were selected. Two independent researchers blinded to the clinicopathological parameters and patients' outcome scored all samples of the gallbladder tissue used in this study. An experienced pathologist evaluated any discrepant cases.

### Cell culture, lentivirus-mediated RNA interference and transfection *in vitro*

Human GBC cell lines (GBC-SD, NOZ, OCUG-1 and SGC-996) were used in this study. The GBC-SD (from the Type Culture Collection of the Chinese Academy of Sciences, Shanghai, China) and NOZ (gift from Professor Liu YB) cells were maintained in DMEM (Corning, USA) supplemented with 10% FBS (Ausbian, Australia), while the OCUG-1 (gift from Professor Liu YB) and SGC-996 (gift from Professor Yang YQ) cells were maintained in RPMI-1640 medium (Gibco, USA) supplemented with 10% FBS, respectively. All of the cell lines were incubated in a CO_2_ incubator (SANYO MCO-175, Japan) at 37°C in a 5% CO_2_ atmosphere.

In order to facilitate stable knockdown of endogenous RNF125 in GBC cells, three short hairpin RNAs (shRNA for RNF125) were designed using the following sequences: 5′-GAA TCA CTC GAA CAC CAC ATA-3′ (KD1), 5′- CCG TTT AAT ACC CGA TGA GAA-3′ (KD2) and 5′-CTGTCCACTTTGCCGTTTAAT-3′ (KD3). The negative control shRNA sequence was 5′-ATC ACT CGA ACA CCACATA-3′. The synthesized DNA oligonucleotides containing these sequences were inserted into the GV115 vector with green fluorescent protein (GFP; Genechem, Shanghai, China), then transfected into above GBC cells. Genechem produced recombinant lentiviruses expressing RNF125- shRNA and the negative control shRNA. The lentivirus carried a copy of GFP and infection efficiency was assessed based on the numbers of GFP-expressing cells using florescence microscopy. The expression of RNF125 in the infected cells was determined by qRT-PCR and western blot analyses. Empty vector-transfected cells were used as control. Primer sequences for vectors construction (GV115-RNF125) are shown as following: Forward: 5′-CCG GGA ATC ACT CGA ACA CCA CAT ACT CGA GTA TGT GGT GTT CGA GTG ATT CTT TTTG-3′, Reverse: 5′-AAT TCA AAA AGA ATC ACT CGA ACA CCA CAT ACT CGA GTA TGT GGT GTT CGA GTG ATTC-3′.

### Microarray analysis *in vitro*

Microarray analysis was performed using the GeneChip® PrimeView™ Human Gene Expression Microarray (Affymetrix, USA). Briefly, total RNA was extracted in triplicate from highly aggressive NOZ and lowly aggressive SGC-996 GBC cells (NOZ *vs*. SGC-996) or from NOZ cells transfected with shRNF125 (knockdown, KD) and the negative control shRNA (NC) (KD *vs*. NC). The quality of the RNA samples was assessed using Nanodrop 2000 (Thremo Fisher, USA), 2100 Bioanalyzer and the RNA 6000 Nano Kit (Aglient, USA). The 100-μg RNA samples were mixed with a poly-A RNA control and processed into double-stranded cDNA. *In vitro* transcription (IVT) of cRNA was then performed by adding 130 μl of IVT Master Mix using a GeneChip 3′IVT PLUS Kit (Affymetrix) to 130 μl of double-stranded cRNA. The generated cRNA was purified, quantified and labeled. Finally, arrays were hybridized in a GeneChip Hybridization Oven 645 (Affymetrix), washed in the GeneChip Fluidics Station 450 (Affymetrix) using a GeneChip Hybridization Wash and Stain Kit (Affymetrix) and scanned using a Genechip Array scanner 3000 (Affymetrix).

Array data were normalized using log scale robust multi-array analysis and were analyzed by R-Project software. Gene expression was deemed significant the fold change (FC) value was > 3.0 (NOZ *vs*. SGC-996) or > 2.0 (RNF125: KD *vs*. NC) and *P* < 0.05. GO was used to perform functional enrichment analysis, which included analyses of biological processes, cellular components, and molecular function. For statistical analysis of GO, gene set enrichment analysis and Fisher exact analysis were performed. As part of this study for gene expression profile variance between NOZ cells and SGC-996 cells, potential GBC invasion-metastasis related genes involved in the biological processes were selected for verification. Furthermore, as part of this study for knockdown of RNF125 in NOZ cells, potential downstream genes involved in the biological processes were selected for verification, and gene interaction networks were constructed using the Reactome database [48].

### Invasion assay *in vitro*

Cell invasion *in vitro* was assessed using the Transwell chambers (Corning, USA). Cells were divided into shCtrl group and shRNF125 group. 100 μl suspensions (1×10^5^ cells/well) were seeded onto the apical chamber in fresh serum-free culture medium, simultaneously with a MTS 96-well plate (Promega, USA; 5 × 10^4^ cells/well) as an invasive reference. Basoleternal chamber was added with 600 μl 30% FBS (Austine). After 24-h in a humidified incubator at 37°C with 5% CO_2_, cells that invaded through the basement membrane were stained with Giemsa (Sigma), counted under an inverted light microscope (Caikang XDS-100, Shanghai, China) or a fluorescence microscope (Olympus IX71, Japan) in 5 independent fields at ×200 magnification. Three independent experiments were performed.

### Migration assay *in vitro*

Cell migration *in vitro* was determined using a wound-healing assay with HCS. Cells were divided into shCtrl group and shRNF125 group. 100 μl cell suspensions (5×10^4^cell/well) were seeded in 96-well wounding plate (VP, USA) with culture medium for 24 h at 37°C in 5% CO_2_ incubator. When cells were cultured in a single layer with 90% confluence, a scratch tester was used to scratch a wound at the central bottom of 96-well plate. Cells were washed thrice with serum-free culture medium to remove the suspended cells, and added with 0.5% FBS for 24 h at 37°C with 5% CO_2_. The cell migrating area was scanned and analyzed at 0 h, 8 h and 24 h using a Cellomocs (Thermo Fisher), and observed under an inverted light microscope (Caikang XDS-100) or a fluorescence microscope (Olympus IX71) at 50 magnifications. Cell migration area (pixel area) = (S3+S4) − (S1+S2). All experiments were performed in triplicate.

### Adhesion assay *in vitro*

A 96-well plate (Coster, USA) was coated with an artificial basement membrane gel i.e., Matrigel (Corning; 2 μg/50μl), then washed with serum-free culture medium. Cells divided into shCtrl group and shRNF125 group were seeded onto the 96-well plate at a density of 4 × 10^3^ cells per well in an incubator for 40 min at 37°C with 5% CO_2_. The unattached cells were removed by tapping the plate and by rinsing the wells with PBS triplicate. Attached cells were counted using a Celigo (Nexcelom, USA) in five representative high-power fields. Three independent experiments were performed.

### QRT-PCR analysis *in vitro*

A total of 47 genes including 39 metastatic-related differentially expressed genes selected from microarray analysis for NOZ and SGC-996 cells (NOZ *vs*. SGC-996), a target gene RNF125, and 5 RNF125 downstream TGF-β1 signaling pathway genes from microarray analysis for NOZ cells with RNF125 knockdown (KD *vs*. NC) were determined by qRT-PCR assay. QRT-PCR was performed as described by the Genechem manufacturer (Shanghai, China). Total RNA was isolated from cultured cells using Trizol reagent, then reverse transcribed into cDNA using M-MLV reverse transcriptase (Promega). The reaction was carried out using SYBR Premix Ex Taq (Takara, Japan) and the Real Time PCR System (Agilent). MRNA qPCR Primer Set of 39 metastatic-related genes were shown in Ribobio (China; http://www.ribobio.com/). The gene-specific primer sequences of RNF125, 5 TGF-β1 signaling pathway genes and the housekeeping gene GAPDH were showed as following: RNF125 (bp194): forward: 5′-CTG AGT GTG ACA CCC TGG TTT-3′, reverse: 5′-CCG TTC CGA TCT GTG ATG AGT-3′; THBS1 (bp157): forward: 5′-AGA CTC CGC ATC GCA AAG G-3′, reverse: 5′-TCA CCA CGT TGT TGT CAA GGG-3′; FST (bp260): forward: 5′-GGA ACT GCT GGC TCC GTC AA-3′, reverse: 5′-GCA GCG GGG TTT GTT CTTCT-3′; ID1 (bp143): forward: 5′-GTA AAC GTG CTG CTC TAC GAC ATGA-3′, Reverse: 5′-AGC TCC AAC TGA AGG TCC CTGA-3′; SMAD3 (bp169): forward: 5′-CCT TTC AGG TAA CCG TCTT-3′, reverse: 5′-TTT AGC CCA TCA TCTC CC-3′; CDKN2B (bp114): forward: 5′-CTG GAC CTG GTG GCT ACG-3′, reverse: 5′-ACA TTG GAG TGA ACG CAT CG-3′; GAPDH (bp121): forward: 5′-TGA CTT CAA CAG CGA CAC CCA-3′, reverse: 5′-CAC CCT GTT GCT GTA GCC AAA-3′. The reaction conditions that were applied to facilitate the generation of cDNA were as follows: 1 cycle at 95°C for 30 s, 45 cycles of a two-step PCR at 95°C for 5s and 60°C for 30 s, and 1 cycle at 95°C for 15 s, 55°C for 30 s and 95°C for 15 s. The mRNA expression of the gene was normalized using GAPDH and calculated using the 2^−ΔΔCt^ methods. Each experiment was independently performed in triplicate.

### Western blotting *in vitro* and *in vivo*

A total of 11 gene proteins including RNF125 protein from 10 fresh GBC and adjacent normal matched specimens and from NOZ cells treated with shRNF125 and shCtrl, 4 EMT markers E-cadherin, vimentin, N-cadherin and β-catenin, and 6 TGF-β1 signaling pathway proteins from NOZ cells treated with shRNF125 and shCtrl were determined by western blot analysis, respectively. Total protein was isolated from tissues or cells using RIPA Lysis Buffer (Genechem), and then determined with a BCA protein assay kit (Biyuntian, China). An aliquot of 20 μg of proteins was subjected to 12% sodium dodecyl sulfate-polyacrylamide gel electrophoresis, transferred to a polyvinylidene difloride membrane (Millipore, USA). An hour after being blocked with TBST containing 5% skimmed milk, the membrane was added in order with each primary antibody [rabbit anti-RNF125 (1:500∼1000), mouse anti-E-cadherin (1:500), anti- rabbit N-cadherin (1:200), anti-mouse THBS1 (1:500), anti-goat FST (0.3–1μg/ml), anti-mouse SMAD3 (1:200–1000), anti-rabbit ID1 (1:1000–2000), anti-rabbit TGF-β1 (1:500), anti-rabbit CDKN2B (1:500–1000) (all from abcam, UK), rabbit anti-vimentin (1:500) and rabbit anti-β-catenin (1:500)(all from CST, USA), anti-rabbit anti-mouse GAPDH (1:2000; Santa-Cruz, USA)] diluted with PBST, and with an appropriate secondary antibody (anti-rabbit IgG or anti-mouse IgG, 1:5000, Santa Cruz; anti-goat IgG, 1:5000, Biyuntian) according to the manufacturer's instructions. The target proteins were visualized using an enhanced chemiluminescent (ECL) reagent (Thermo Fisher); and the signal was recorded using photographic film. The gray value and gray coefficient ratio of every protein were quantified using Quantity One software. GAPDH expression was used as internal controls.

### Statistical analysis

All data were expressed as mean ± SD and analyzed using SPSS (22.0 version software, IBM, USA). Statistical analyses to determine significance were tested with the two-tailed Student's *t*-test and χ^2^ test. Survival curves were calculated with the Kaplan-Meier method and were compared using the log-rank test. Univariate and multivariate analyses of prognostic factors were performed using the Cox's proportional hazards regression model. *P* < 0.05 was considered statistically significant.

## Abbreviations

RNF125: ring finger protein 125
TGF-β: transforming growth factor-β
SMAD3: SMAD family member 3
ID1: inhibitor of differentiation and DNA binding-1
CDKN2B: cyclin-dependent kinase inhibitor 2B
THBS1: thrombospondin1
FST: follistatin
AEG-1: astrocyte elevated gene-1
HIF-1α: hypoxia inducible factor-1α
SPOCK1: sparc/osteonectin, cwcv, and kazal-like domains proteoglycan 1
PI3KCA: phosphoinositide 3kinase CA
PI3K/AKT: phosphoinositide 3kinase/focal adhesion kinase
PI3-K/MMPs/Ln-5γ^2^: phosphoinositide 3 kinase/matrix metalloproteinases/ laminin 5γ^2^
EphA2/FAK/Paxillin: ephrin type a receptor 2/focal adhesion kinase/ Paxillin
EMT: epithelial-mesenchymal transition
MET: mesenchymal-epithelial transition
IHC: immunohistochemistry
DAB: 3, 3-diaminobenzidine
FBS: fetal bovine serum
PBS: phosphate buffer saline
DMEM: Dulbecco's modified Eagle's media
ECM: extracellular matrix
shRNA: short hairpin ribonucleic acid
GFP: green fluorescent protein
HCS: high content screening
qRT-PCR: quantitative (real time) reverse transcription-polymerase chain reaction
GAPDH: glyceraldehyde-3-phosphate dehydrogenase
OS: overall survival
UICC: Union for International Cancer Control.
